# Effects of a New Type of Grinding Wheel with Multi-Granular Abrasive Grains on Surface Topography Properties after Grinding of Inconel 625

**DOI:** 10.3390/ma16020716

**Published:** 2023-01-11

**Authors:** Adrian Kopytowski, Rafał Świercz, Dorota Oniszczuk-Świercz, Józef Zawora, Julia Kuczak, Łukasz Żrodowski

**Affiliations:** 1 Institute of Manufacturing Technology, Warsaw University of Technology, 00-661 Warsaw, Poland; 2 Faculty of Chemistry, Warsaw University of Technology, 00-661 Warsaw, Poland; 3 Faculty of Materials Science and Engineering, Warsaw University of Technology, 00-661 Warsaw, Poland

**Keywords:** grinding, multigranular wheels, abrasive grains, Inconel 625, response surface methodology

## Abstract

Finishing operations are one of the most challenging tasks during a manufacturing process, and are responsible for achieving dimensional accuracy of the manufactured parts and the desired surface topography properties. One of the most advanced finishing technologies is grinding. However, typical grinding processes have limitations in the acquired surface topography properties, especially in finishing difficult to cut materials such as Inconel 625. To overcome this limitation, a new type of grinding wheel is proposed. The tool is made up of grains of different sizes, which results in less damage to the work surface and an enhancement in the manufacturing process. In this article, the results of an experimental study of the surface grinding process of Inconel 625 with single-granular and multi-granular wheels are presented. The influence of various input parameters on the roughness parameter (Sa) and surface topography was investigated. Statistical models of the grinding process were developed based on our research. Studies showed that with an increase in the cutting speed, the surface roughness values of the machined samples decreased (Sa = 0.9 μm for a *Vc* of 33 m/s for a multigranular wheel). Observation of the grinding process showed an unfavorable effect of a low grinding wheel speed on the machined surface. For both conventional and multigranular wheels, the highest value for the Sa parameter was obtained for *Vc* = 13 m/s. Regarding the surface topography, the observed surfaces did not show defects over large areas in the cases of both wheels. However, a smaller portion of single traces of active abrasive grains was observed in the case of the multi-granular wheel, indicating that this tool performs better finishing operations.

## 1. Introduction

As a result of the developments in the field of material technology, there is a strong need to produce new alloys. These materials are mainly characterized by properties such as creep resistance or heat resistance, thanks to which they are widely used [1,2]. Ezugwu et al. [3] indicated that these properties allow them to be widely used in advanced designs of aircraft engines, as well as gas turbines and spacecraft. Due to their mechanical properties, nickel-based superalloys such as Inconel 625 belong to the group of difficult to cut materials. Evans et al. [4] suggested that the presence of the elements C, Cr, Mo, and Nb increases the mechanical and thermal properties of the material. The studies undertaken by researchers are mainly focused on shaping the geometry, based on the use of unconventional machining technologies such as EDM [5,6,7], electrochemical machining [8,9], or hybrid machining [10,11]. An integral part of technology development is also the use of finishing treatments in the process of shaping a specific state of the surface layer [12,13,14,15]. The treatment under consideration in terms of surface finish is grinding. Jackson and Davim [16] described grinding as an alternative to the non-traditional machining process. They justified it especially with regards to manufacturing hardened materials and elements requiring high precision due to the control of residual stresses and microstructural changes. The development of grinding processes is primarily influenced by increasingly higher requirements for the quality of surface finish. On the other hand, the grinding process, according to Ruzzi et al. [17], shows peculiarities in relation to machining processes with geometrically well-defined cutting edges, such as milling operations. In addition, the emergence of more modern construction materials with difficult to machine properties should be highlighted. Pratap et al. [18] noticed more and more higher requirements concerning the quality of cutting tools. This has led to the construction of innovative machine tools, which are mainly based on new kinematic varieties of grinding processes. Batham et al. [19] reviewed the latest advances in micro-grinding tool manufacturing techniques. They noted that tool life and surface finish are the main problems where micro-grinding hard and brittle materials is concerned. The grinding process of Inconel 625 is difficult due to the low thermal conductivity of the material. The limited heat dissipation from the machining zone also adversely affects the machining tool itself, reducing its lifetime. As a result, dimensional and shape inaccuracies in the workpiece arise. In the area of finishing, attention is drawn to the trend of creating innovative abrasive tools based on new materials. Souza et al. [20] proposed a coordinated analysis that incorporated traditional parameters and sustainability criteria (economic, environmental and social) that are feasible and proven effective, enabling better decision making on grinding wheel performance. Grinding, which is the basic representative of the group of finishing operations, is primarily responsible for giving integral features to the mating surfaces. The development of technology affects the cyclical increase in machining efficiency. The discussed process draws attention to the method of intensifying machining, consisting of the formation of a properly developed profile of the active surface of the grinding wheel. According to Jourani et al. [21], the influence of local abrasive grain geometry on temporary contact parameters, such as the coefficient of friction and wear rate, has not yet been fully elucidated. Experimental research on the grinding process focuses mainly on determining the effects of machining in relation to the input parameters used. Zhao et al. [22] distinguished between three modes of grain wear: (I) microcracks that appear mainly in the initial stage of wear; (II) abrasive wear, which corresponds to the stage of constant wear; and (III) macrocracks and tears that occur in the phase of heavy wear under high shear loads. According to this line of reasoning, the duration of the permanent wear phase has a large impact on the service life and efficiency of grinding [23,24]. Li et al. [25] proposed an analytical model from a microscopic perspective (including friction force and cutting force of a single grain). However, the development of grinding force modeling, taking into account the actual grinding wheel topology and the non-deformed chip thickness heterogeneity, still has great potential. In the grinding process, surfaces are treated where machining allowances have been left in the previous treatment. The left material sizes are often mechanically and thermally damaged. Destruction of the surface caused by grinding directly affects the dimensional accuracy of the processed element, the technical condition of the surface layer and the change in the tribological characteristics of the active abrasive surface. The observations on scratch morphology noted by Hmadi et al. [26] prove that one abrasive grain causes several scratches on the machining surface. The machining zone is a place where variable distributions in unit pressures, velocities of relative deformations of the removed material and temperature occur. According to Kacalak et al. [27], the geometrical features of the abrasive grains and their orientation in the cutting direction significantly affect the material removal process, which in turn affects the topography of the treated surfaces. During micro-cutting processes, the tips of active grains wear and break, which affects the profile of machining marks and the material separation mechanisms.

To date, the research on the grinding process relates to both physical analysis of the process and determination of the correlation between the process parameters and the roughness and geometrical accuracy of the surface [28].

A relatively small number of studies are related to the development of new tools that could significantly change the micro-cutting process. Existing publications in the area of abrasive tool construction deal with interference with its design by making notches on the active surface [29], segmented construction [30], or discontinuities and modifications of porosity size [31]. Researchers have also analyzed the effect of grinding wheel texturing on the grinding process [32]. Texturing can also take place during dressing of the grinding wheel [33].

Sustainable technological development involves improving the conditions of micromachining by conducting roughing and finishing in a single pass [34]. In addition, it improves the quality of the achieved surface of machined workpieces. This is especially important in the case of finishing operations of difficult to cut materials such as Inconel 625, which is widely used in the aerospace and energy industries [35]. Overcoming the limitations in grinding efficiency to obtain the desired surface topography properties is quite a challenging task. Therefore, in this study a new type of grinding wheel with grains of different sizes (multigranular wheel) is proposed. The following research is aimed at a complementary use of a multigranular wheel in relation to the obtained surface topography properties. The results were compared with classic abrasive (single-granular) wheels. Unlike other researchers, we determine regression equations for the Sa parameter, which describes the relationship between input and output data. The obtained results are distinguished by the use of a new type of abrasive disc, which was additionally compared with a conventional one. In practical terms, the presented research and results analysis enabled defining the most advantageous parameters of the grinding process.

## 2. Materials and Methods

In this research, attention was mainly focused on the influence of the micro-cutting process of abrasive grains on the surface topography properties during grinding Inconel 625. Some properties of Inconel 625, such as good high-temperature creep and excellent oxidation resistance and corrosion resistance derived from the addition of molybdenum and niobium to the basic nickel-chromium alloy composition, allow a wide range of applications in the aerospace or energy industries [36,37]. However, its chemical composition and properties make it difficult to process. For that reason, new solutions are sought, which would enable obtaining the desired features of the surface topography in the grinding process. The chemical composition of Inconel 625 is presented in Table 1.

**Figure 1:**
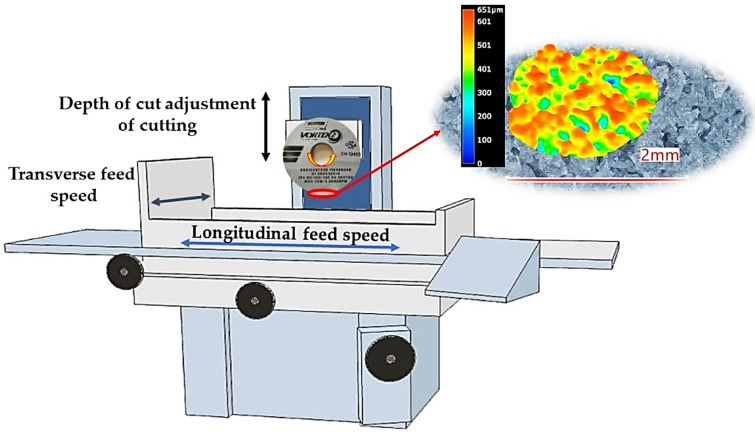
Scheme of the machining station.

Experimental investigation of the influence of cutting speed, *Vc*, transverse feed speed, *Vp*, and longitudinal feed speed, *Vw*, on the roughness parameter Sa and surface topography properties was conducted according to the Design of Experiment (DOE) methodology. A five-level rotatable DOE, in which there are experiments in the stellar arms, equidistant on each axis by a value of +/− α, was chosen. The plan was supplemented with three repetitions in the central point (0,0,0). The experiment was performed on a Jotes SPC20b (Figure 1) surface grinder (the station was expanded with controllers determining the feed speed and spindle speed control).

Inconel 625 samples with dimension of 38 × 8 × 5 mm were used during the grinding process. Typical grinding parameters used in the experiment, such us cutting speed (*Vc*), transverse feed speed (*Vp*) and longitudinal feed speed (*Vw*), were determined according to preliminary research and a review of the literature [39]. Process parameters such as machining speed and feed rate are the key operational input factors that determine the output parameters such as surface roughness, tool wear and surface damage [39]. For the presented ranges, it was possible to observe changes in the geometric structure of the surface. Parameters such as cutting speed and feed rate are the main factors influencing the grinding process. The experimental conditions and range of investigated grinding parameters in the DOE are presented in Table 2 and Table 3, respectively.

In the conducted experimental studies, two types of abrasive grinding wheels with dimensions of 250 × 32 × 76 mm from Norton Saint-Gobain (Conflans-Sainte-Honorine, France) were used: a conventional wheel with a granulation of 100, and a new type multigranular with a mixture of granulation abrasive grains 80/100/120. The tools used are dedicated to the machining of Inconel 625, among others. The use of a new type of abrasive tool (multigranular wheel) would enable the intensification of the machining process and the achievement of the desired characteristics of surface topography [40]. Special attention was focused on establishing the influence of technological parameters of the grinding process of Inconel 625 on selected technological indicators.

The abrasive wheels used are constructed of green silicon carbide with a ceramic bond. The grinding wheel material used is characterized by the content of abrasive grains (rhombohedral) with very sharp edges, which are harder than the grains of electro-corundum [41,42].

The research began with tests with a conventional grinding wheel, 01_250x32x76_IPA_100_HA_26VTX2. The sample was mounted in a precision vice on a magnetic grinding table. Then, the input parameters were set on the grinder, i.e., cutting speed (*Vc*), longitudinal feed speed (*Vw*) and transverse feed speed (*Vp*), resulting from the adapted experimental plan. Before machining, for every sample the test was performed with a new surface of grinding wheel; each time the wheel was dressed before manufacturing. For this purpose, a MKOx25 dresser with a PCD plate was used. With the tool prepared in this way, the machining process was started (with previously set machining parameters). The set depth of cut was 0.03 mm. Performing all the tests from the adopted plan ended the first stage of the works. In the next step, a multigranular wheel, 01_250x3 x76_IPA_80/100/120_HA_26VTX2, was installed on the machine. Tests were repeated according to the above procedure.

The surface topography of the grinding samples was measured with a scanning profilometer from Taylor Hobson (Form Talysurf Series 2) (Taylor Hobson, Leicester, UK). An area of 2 × 4 mm was measured with a discretization step of 10 μm on the Y axis. The topography properties were described using the following parameters:

- Sa: arithmetic mean of the deviations from the mean Sa (average value of the absolute heights over the entire surface);
- Surface topography.

Observation of the grinding wheel topography and sample topography was performed with a Keyence VHX Microscope (Japan) and a Hitachi SEM Microscope (Japan), respectively.

The response surface methodology was used to build the prediction models of the grinding process. In this paper, regression equations were determined, which are described by a second-degree polynomial function (showing the relationship between cutting speed, longitudinal feed rate, transverse feed rate and the roughness parameter, Sa) [43].

## 3. Results and Discussion

### 3.1. Analysis of the Grinding Wheel’s Surface Topography and Sample Properties

**Figure 2:**
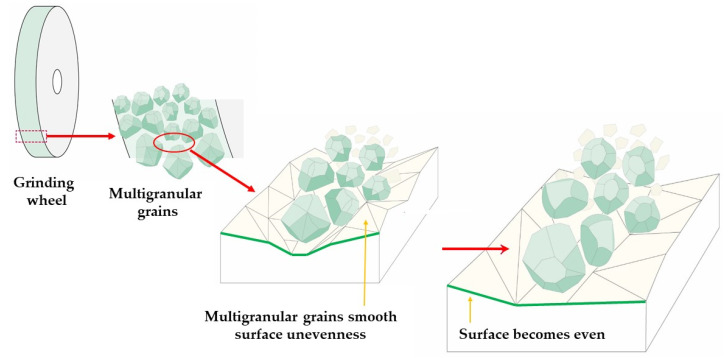
Mechanism of multigranular wheel’s work.

Grinding wheel surface topography properties are one of the main factors which have a strong influence on the abrasive grain micro-cutting process and sample topography properties after finishing [44]. However, high requirements for grinding tools have been implemented to improve their productivity by reducing tool wear and avoiding grinding burn [45,46]. The steady wear stage must be extended and the rapid failure of grinding wheels should be avoided in order to achieve the desired grinding efficiency and wear-resistant properties. However, the grinding efficiency and tool life are severely affected by the possible macro-fracture and pull-out of grains. A series of studies have demonstrated the effects of grinding parameters on material removal behavior, and have suggested measures to find the most advantageous parameters of the grinding process. The grinding wheel properties are mainly affected by the abrasive material and the binder. Wang et al. [47] studied the influence of the grinding wheel granularity on the grinding force and material removal. They found that with an increasing granularity of the grinding wheel, the grinding amount and surface roughness of the materials decreased. It was noted that the grinding heat originated mainly from the abrasive grain cutting behavior. Thus, in the present study, the influence of the effect of a multi-granular abrasive wheel on grinding behaviors will be investigated. Due to the high wear and tear of grinding wheels during the process, it is important to optimize the abrasive wheel design; hence, a new wheel type consisting of several abrasive grain sizes was designed in the study [48,48]. Figure 2 shows a scheme of how the use of a multigranular wheel results in a better surface topography. The conventional grinding wheel is characterized by a deeper step depth which results in greater surface roughness [49]. Unlike a conventional wheel, a multi-granular wheel contains grains of different sizes. This design results in better filling of the active surface of the grinding wheel, because the spaces between the larger grains are filled with smaller fractions. Such a construction provides an opportunity to reduce the chipping off of grains and reduce the grinding wheel wear by reducing elastic impacts. Another advantage of a multigranular grinding wheel is the possibility of forming a smaller micro-grit, and thus a better drainage, reducing the surface damage. As a result, less force is exerted on a single abrasive grain, and the obtained surface is characterized by lower roughness. In the opposite case, when a multigranular wheel is used, small abrasive grains are packed between bigger ones, which prevents chipping and obtunding and results in lower surface roughness.

**Figure 3:**
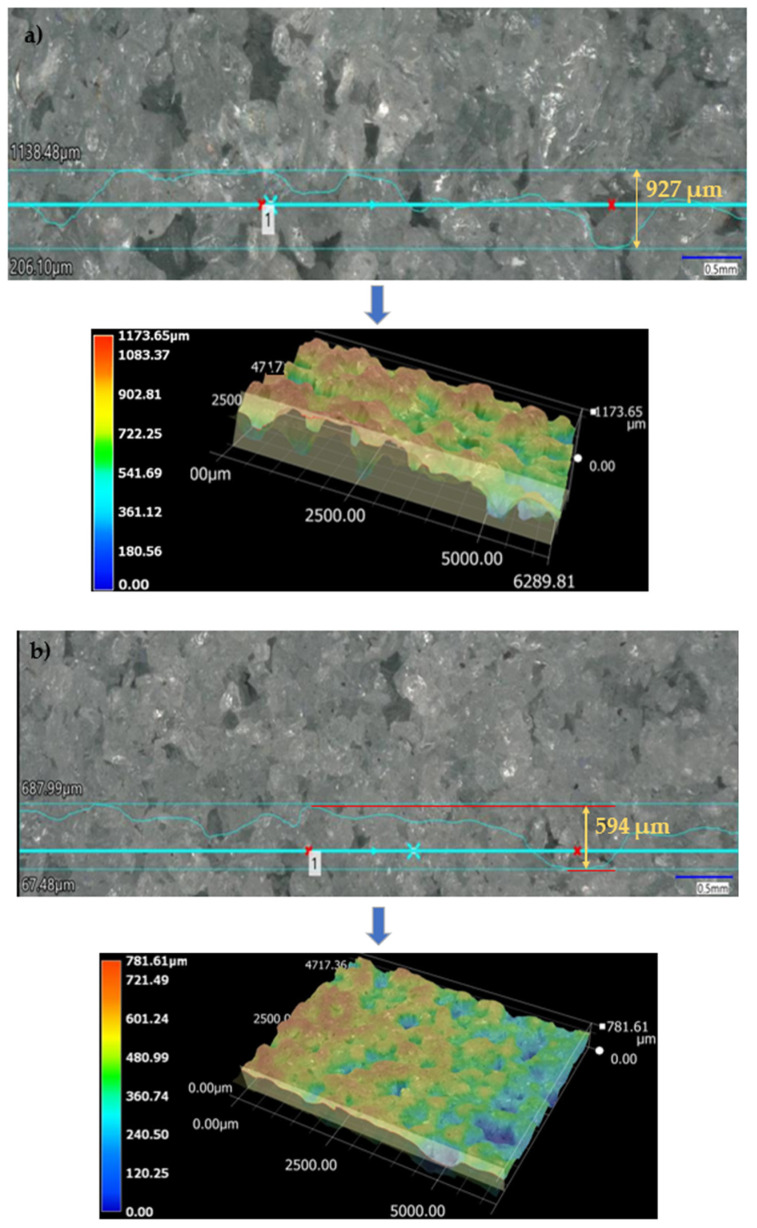
Active surface area of (**a**) a conventional and (**b**) a multigranular grinding wheel.

This study showed a significant effect of the type of abrasive wheel on the quality of grinding. Figure 3 shows images of the active surface structures of a conventional (Figure 3a) and multi-granular (Figure 3b) grinding wheel [50,51]. The grains of the conventional wheel had larger height amplitudes (927 µm) and smoother cutting edges. The negative angle of attack of these grains was much smaller, so that these grains tended to form chips of typical burr topography [52]. The cutting of the abrasive grains into the surface of the sample caused plastic flow. During grinding, the grains on the surface were continuously worn away. On the other hand, the multigranular wheel showed higher grain fracture toughness. The physics of the grinding phenomenon shows that there was increased grain chipping in the conventional disc, showing abrasive wear [53]. With regard to the quality of grinding, the conventional wheel had a greater depth of abrasive grains micro-cuts, resulting in an increase in surface roughness compared to the multigranular disc [54].

**Figure 4:**
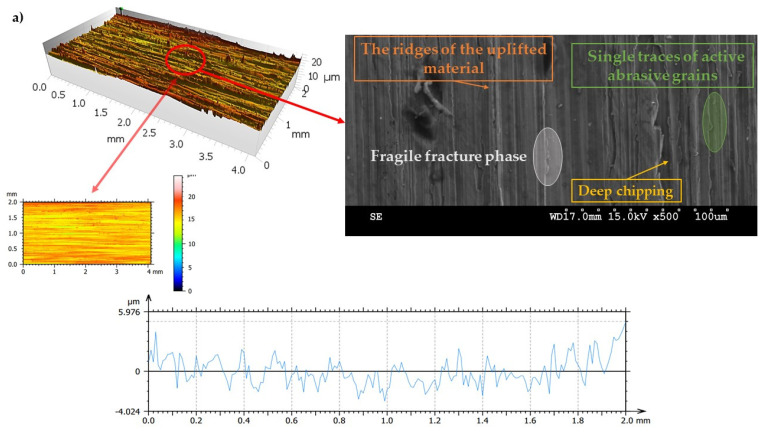

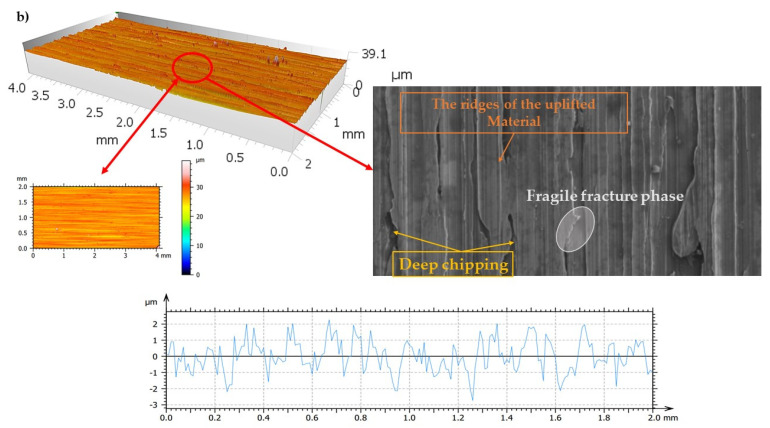
Surface texture of the specimen after machining with (**a**) a multigranular wheel and (**b**) a grinding wheel. *Vc* = 33 m/s, *Vp* = 133 mm/min and *Vw* = 13,900 mm/min.

In order to characterize the properties of the treated surfaces in the process of grinding with a conventional and a multi-granular grinding wheel, a comparative analysis of the surface structure features was performed for sample 4 (DOE), for which the lowest Sa roughness was obtained. The sample surface topography analysis after grinding indicates that during machining, furrowing of the machined material was observed (Figure 4). This is characteristic of the grinding process in a plastic flow situation [55]. The observed surfaces do not show defects over large areas, which is characteristic of brittle fracture mechanisms. The samples show single traces of active abrasive grains, located in the active zone of the grinding wheel. The smaller proportion of cracks for the multigranular wheel (Figure 4a) indicates that the tool is conducting roughing and finishing operations. As a result of a detailed analysis of the resulting traces, one can see a resemblance to surfaces shaped in the process with selected abrasive grains. The work of individual abrasive grains results in the formation of a pile-up of material between them, with a characteristic longitudinal course, which occurs as a result of plastic flow [56]. A similar effect was observed by Wang et al. [57]. The tops of the resulting rises are often jagged. Images attributed to an intermediate phase between brittle and plastic flow of material are observed. A smaller proportion of ridges in the multigranular wheel indicates a better ability of the tool to cut off the chip. As a result of the grinding process, deep machining cracks were also observed in the sample. Observation of the resulting pits did not show the characteristic damage of a brittle fracture. As a result of machining with a conventional grinding wheel, deep scratches penetrated by abrasive grain can be seen (Figure 4b).

The surface topography after the grinding process for specimens machined with a multi-granular wheel and a conventional grinding wheel exhibits a complex structure [58]. To describe the surface properties, the following functional parameters were chosen: the arithmetic mean of the deviations from the mean Sa (the average value of the absolute height over the entire surface), Sk (the roughness of the core), Spk (the roughness of the peak) and Svk (the roughness of the valleys). The roughness parameters, Sk, Spk and Svk, describe the load capacity of the surface (Table 4). The roughness parameter, Svk, and that of the lower bearing surface (Smrk2) give information about the surface lubrication properties, i.e., the ability of fluid to flow through the sliding surfaces [59]. The roughness of the peak (Spk) can give information on the surface’s resistance to abrasion. The higher the Spk value, the lower the resistance to abrasion. The roughness of the core (Sk) determines the depth of the roughness after the initial breaking-in period.

The Spk parameter after grinding Inconel 625 was equal to 1.1 and 1.8 in the case of the multigranular and conventional wheels, respectively. Accordingly, Svk parameters were measured as 1.6 and 2. The change in wheel had a significant influence on surface topography. After use of a conventional wheel, an almost 60% increase in the Spk value can be noticed, in comparison to the multigranular wheel. Moreover, an almost 30% decrease in the Sk value in the case of the multigranular wheel is noted, which is a result of the usage of sample.

### 3.2. Response Surface Methodology

The surface topography properties after the grinding process are generated by the influence of the micro-cuts of abrasive grains. Depending on the investigated grinding process parameters and the type of abrasive wheel used, there were significant differences in surface topography properties. The influence of the three major grinding parameters on the roughness parameter, Sa, was investigated: cutting speed, *Vc*, was in the range of 7–34 m/s, transverse feed speed, *Vp*, was in the range of 6000–16,000 mm/min and the longitudinal feed speed, *Vw*, was in the range of 90–300 mm/min. Experimental research was carried out in accordance with the central composite rotatable design of the experiment with three factors and five levels. The choice of such a type of DOE allowed for the study of a wide range of variability (at five levels) in the influence of selected independent variables on the investigated output. Moreover, the use of DOE enables a reduction in the number of experiments in order to obtain statistically significant results. The results of the experimental studies for conventional and multigranular grinding wheels are presented in Table 5.

The surface roughness, Sa, was in the range of 0.90–4.00 µm for the conventional wheel and 0.90–3.80 µm for the multigranular abrasive wheel. Results obtained for the multigranular and grinding wheel are presented in Table 6. The highest Sa value (>3.4 µm) was acquired for a *Vc* of 7 m/s. This observation, in agreement with the literature, indicates a negative influence of low cutting speed on surface topography. Mean Sa values oscillating within 1.5–2 µm were obtained for a higher cutting speed (13 m/s) and a lower longitudinal feed speed (133 mm/min). The lowest Sa value was acquired for a *Vc* of 33 m/s. In this case, effective cleaning of machining products, preventing congestion of the wheel and an enhancement of the role of the abrasive grains in the active profile of the wheel, can be noticed.

Based on the response surface methodology, regression equations were developed to describe the correlation between the input parameters of the process and the response parameters. A regression analysis with a backward elimination process was performed. For each equation, the correlation coefficient, R, determined the variability in the studied characteristic. It was in the range R ϵ <0, 1>. Obtaining a value of R close to unity gives a better representation of the variability in the studied trait. The adequacy of the obtained coefficient of multiple correlation was verified by the Fisher–Snedecor test. The R-squared coefficients of determination values were calculated. The coefficients represented the percentage of variance explained by the model. After eliminating irrelevant factors in the response equations for surface roughness (Sa), the final form of the developed function is presented in Equations 1 (for a multigranular wheel) and 2 (for a conventional wheel). Table 7 summarizes the regression statistics for the designated equations. According to the theory of the planned experiment, three repetitions were made at the focal point. A total of 17 samples were prepared for the experiment and used to carry out 17 tests for a conventional shield and a multi-granular shield for specific input parameters.

Sa = 9.98 − 0.00096·*Vw* − 0.24·*Vc* + 0.000000037·*Vw*^2^ + 0.0037·*Vc*^2^ + 0.00000047·*Vw*·*Vp* (1)

Sa = 12.81 − 0.00037·*Vw* − 0.039·*Vp* − 0.38·*Vc* + 0.0036·*Vc*^2^ + 0.0000016·*Vw*·*Vp* + 0.00083·*Vp*·*Vc* (2)

The analysis of the residuals for the developed models for Sa confirmed the basic assumptions. The residuals had a normal distribution, a constant variance and were independent of the order of the data. The assumption of constant variance was verified by plotting the residuals against the predicted values. The assumption of normality and independence of the residuals were verified by plotting the expected normal value against the residuals and the residuals against the order of the data, respectively.

The response function was built based on the regression analysis and analysis of variance (ANOVA). For the adapted model of regression function (polynomial of the second degree) at a confidence level of 93%, each independent variable was established. ANOVA results after the elimination of non-significant variables for surface roughness, Sa, are presented in Table 8 and Table 9.

Table 8 shows the ANOVA results for the surface roughness, Sa, obtained with the conventional wheel. The calculated contribution indicates that the greatest influence on surface roughness, Sa, for this disc was the cutting speed (41.6%). The second most influential variable was the product of the transverse feed speed and the cutting speed (24.1%), followed by the square of the cutting speed (16.3%). The contributions of the other variables on Sa were significant but less important. The ANOVA results shown in Table 9 for the multigranular wheel indicate that cutting speed has the greatest effect on Sa (78.9%). This was followed by the square of the cutting speed (12.7%). The contributions of the other variables on Sa were significant but less important.

**Figure 5:**
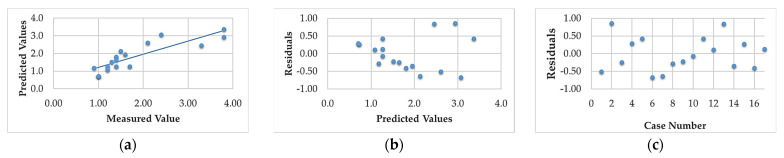
Model checking graphs for surface roughness Sa: (**a**) normal residuals plot; (**b**) residuals versus predicted values; and (**c**) residuals as a function of cases.

Analysis of the normal residuals of the probability plots (Figure 5a) showed that the residuals had normal distributions. Plots of residuals as a function of predicted values (Figure 5b) and residuals versus case number values (Figure 5c) showed that residuals were stochastic. Analysis of the plotted residuals against case values showed that the error terms were independent of each other. Analysis of the residuals confirmed the adequacy of the developed models.

**Figure 6:**
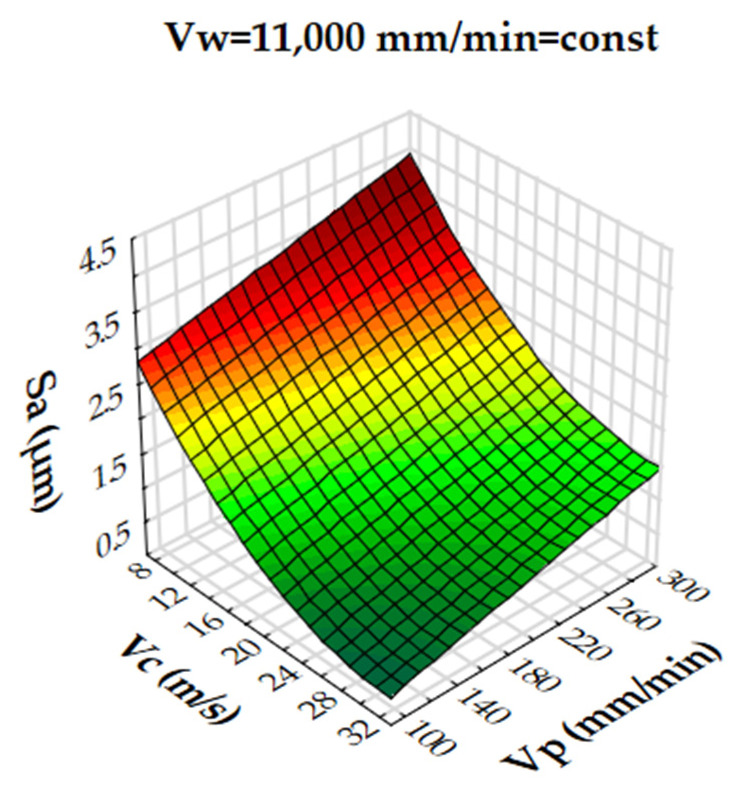
Dependence of the roughness, Sa, on the transverse feed rate, *Vp*, and the cutting speed, *Vc*, for a multigranular wheel (constant *Vw*).

**Figure 7:**
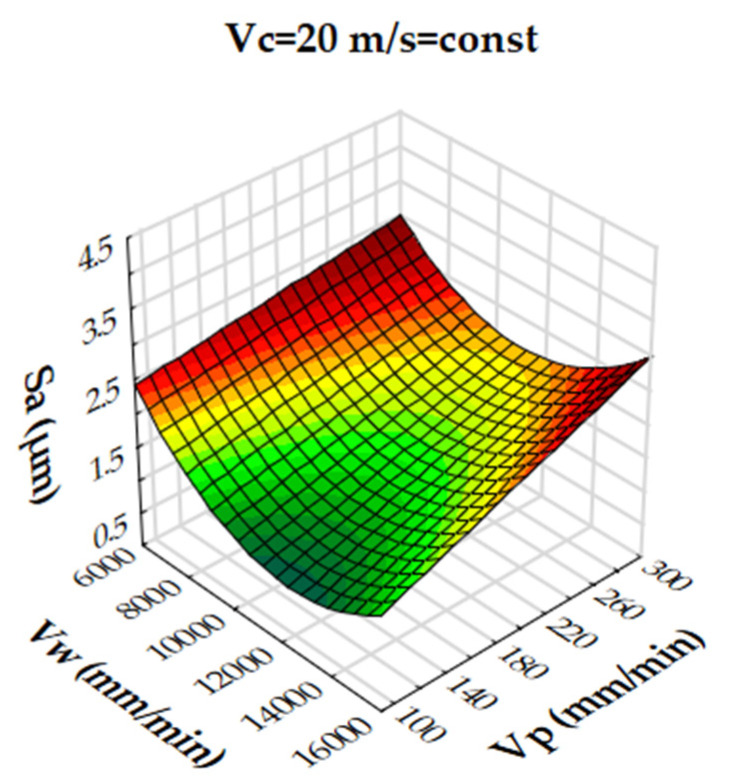
Dependence of roughness, Sa, on longitudinal feed velocity, *Vw*, and transverse feed velocity, *Vp*, for a multigranular wheel (constant *Vc*).

**Figure 8:**
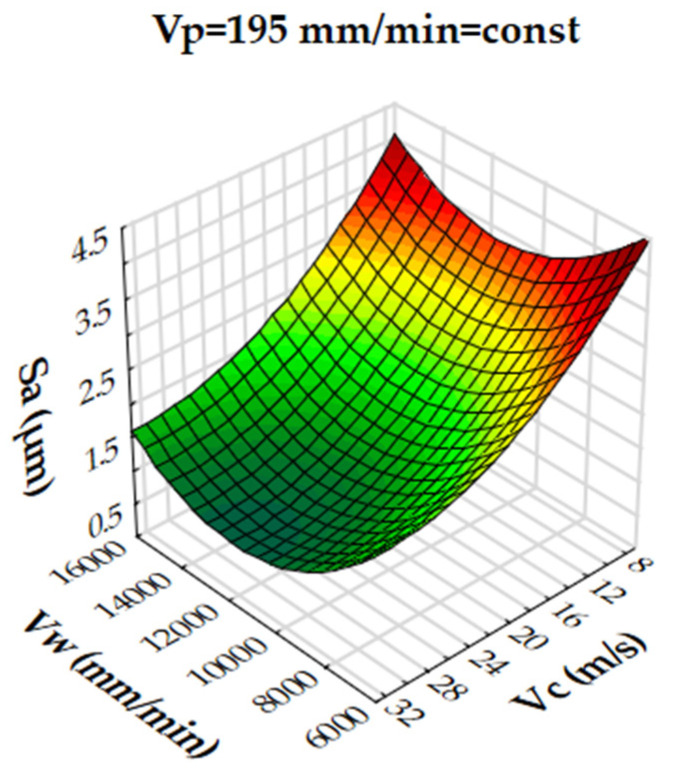
Dependence of roughness, Sa, on longitudinal feed rate, *Vw*, and cutting speed, *Vc*, for a multigranular wheel (constant *Vp*).

**Figure 9:**
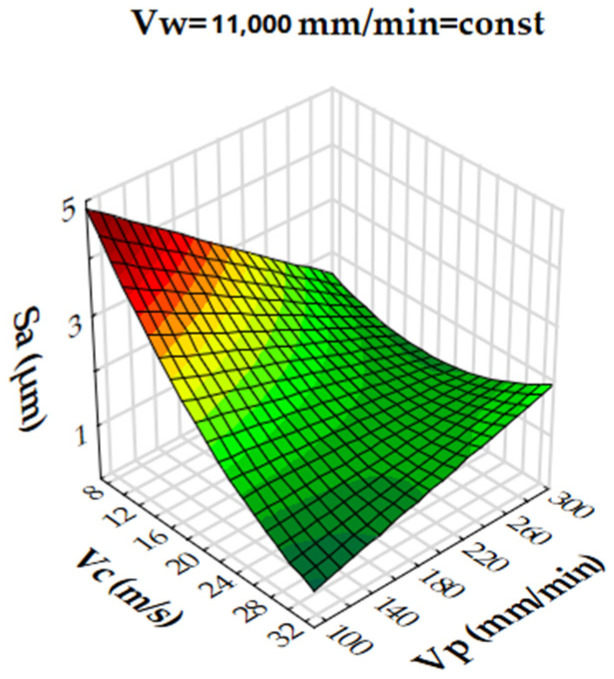
Dependence of roughness, Sa, on transverse feed rate, *Vp*, and cutting speed, *Vc*, for a conventional grinding wheel (constant *Vw*).

**Figure 10:**
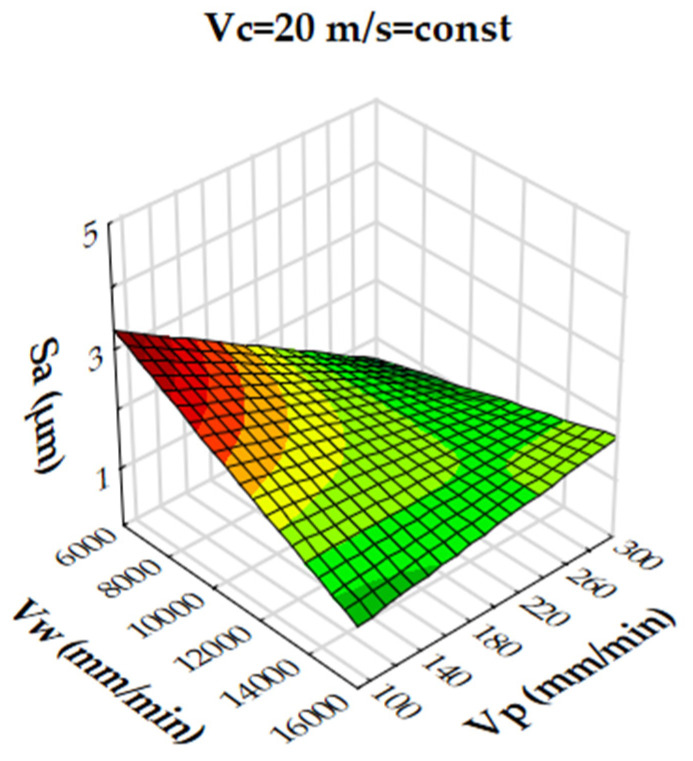
Dependence of roughness, Sa, on longitudinal feed velocity, *Vw*, and transverse feed velocity, *Vp*, for a conventional grinding wheel (constant *Vc*).

**Figure 11:**
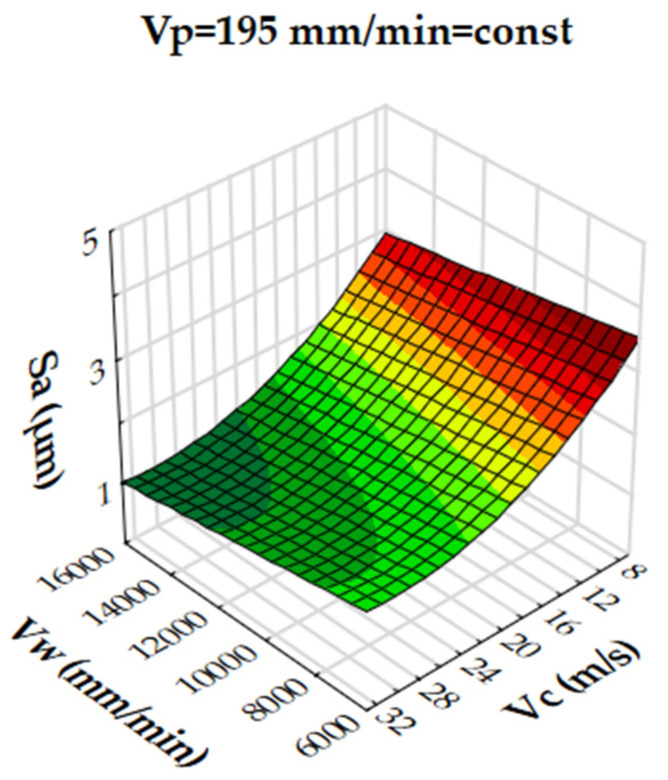
Dependence of roughness, Sa, on longitudinal feed speed, *Vw*, and cutting speed, *Vc*, for a conventional grinding wheel (constant *Vp*).

Analysis of the results showed that the R-squared value for surface roughness (Sa), was more than 86% and 94% for the multigranular wheel and conventional disc, respectively. These results indicate that regression models provide a very good explanation of the relationship between independent variables and Sa response. The differences between R-squared and R-adjusted were less than 0.2, indicating that the established model was adequate to represent the process. The developed models can be used to predict surface roughness values (Sa). Graphical interpretations of the obtained regression equations, describing the studied parameter Sa, are shown in Figure 6, Figure 7, Figure 8, Figure 9, Figure 10 and Figure 11 for a convectional and multigranular grinding wheel, respectively.

The arithmetic mean deviation of the height of surface roughness from the reference plane Sa for the multigranular wheel for the range of machining parameters studied was between 0.9 and 3.8 µm. The obtained range in variation corresponds to the values for roughing and finishing machining. An analysis of the graphs in Figure 6, Figure 7 and Figure 8 indicates that all parameters affect the Sa value. At a constant cutting speed, *Vc*, of 20 m/s, a slight effect of transverse feed, *Vp*, on the roughness value, Sa, is observed (Figure 7). An increase in cutting speed and longitudinal feed speed, *Vw*, results in a decrease in roughness, Sa. This is not a linear relationship, as a local minimum is observed for the longitudinal speed of 11,000 mm/min (Figure 8). The combination of the effect of longitudinal speed and transverse speed also does not show a linear relationship (Figure 7). In Figure 8, one can see that the lowest Sa value is obtained for high *Vc* and medium *Vw* values. On the other hand, a combination of a high cutting speed and a relatively low transverse feed rate results in a low value for the Sa parameter (Figure 6). Increasing the speed of the grinding wheel reduces the contact time of the abrasive grain with the workpiece (during one revolution), which also reduces the grinding force. As a result, a lower surface damage is achieved. Studies determining the influence of input parameters on the geometric structure of the surface enable conduction of very efficient machining. Similar studies conducted by other researchers have provided a basis for many insights [39,60,61].

The arithmetic mean deviation of the height of surface roughness from the reference plane Sa for the grinding wheel for the range of machining parameters studied was between 0.9 and 4 µm. The obtained range in variation, as in the case with the use of a multigranular wheel, corresponds to the values for roughing and finishing. An analysis of the graphs in Figure 9, Figure 10 and Figure 11 indicates that the main parameter affecting the value of the Sa parameter is the cutting speed, *Vc*. With its increase, a decrease in roughness was observed. At a constant cutting speed, *Vc*, of 20 m/s, a slight effect of the transverse feed on the roughness value, Sa, is observed (Figure 10). As observed in Figure 6, a small Sa value is obtained for high *Vc* and low *Vp*. In contrast to the results for the multigranular disc, the situation is different for the juxtaposition of *Vw* and *Vc* (Figure 11). A clear inflection point when changing *Vw* cannot be observed. In such a situation, the abrasive grain wears less at higher *Vp* values. In addition, higher feed values of *Vp* allow more efficient chip removal and disc flooding. This results in lower Sa values (about 1 μm), and for a multi-grain disc it is even below 0.5 μm (due to the reduction in sealing resulting from the disc design). The complementary analysis of grinding parameters in the results obtained makes it possible to look for innovative solutions in other research works [39,60,61].

## 4. Conclusions

This paper focuses on the analysis of the grinding process of Inconel 625 using a new type of grinding wheel. The article takes a comprehensive approach to the grinding process by determining the influence of selected parameters of the grinding process, i.e., the cutting speed and transverse and longitudinal feeds, on selected parameters of surface roughness, Sa, obtained during machining with a new type of grinding wheel. In addition, the results were compared with a conventional disc. The obtained values enabled the derivation of regression equations describing the studied relationships. The purpose of the conducted research was to determine the effect of selected parameters of the grinding process, i.e., the cutting speed and transverse and longitudinal feed rates, on the selected parameters of surface roughness, Sa. The experimental results obtained made it possible to formulate following conclusions:

1. The obtained regression equations describing the studied correlations are characterized by a high correlation coefficient (R for the multigranular disc is equal to 0.87 and for the conventional disc it is 0.92), which indicates a strong relationship between the variables;
2. As the cutting speed, *Vc*, increases, the values of surface roughness, Sa, of the machined samples decrease (to about 0.9 μm, for a *Vc* equal to 33 m/s). Observation of the grinding process indicated the unfavorable effect of the low speed of the grinding wheel on the machined surface. In this case, the particles of the machined material were affected by higher force values during grinding, decreasing the roughness parameters (Sa is close to 3.5 μm for a *Vc* equal to 7 m/s);
3. In some cases, the analyzed values of the Sa parameter differed twice. Such values were obtained for low input parameters, i.e., a *Vw* of 8027 mm/min, a *Vp* of 133 mm/min and a *Vc* of 13 m/s, which is due to the higher load per single abrasive grain in the conventional disc. For a multigranular disc, the active profile of the grinding wheel is more even due to better filling, which results in a reduction in grain-object interactions. Chipping and surface damage are minimized in this way;
4. An analysis of the behavior of the material in the machining zone indicated the various phases of the development of the machining trace (initiation, development and extinction). There are defects on the surfaces obtained by machining with a grinding wheel and a multigranular wheel, indicating the occurrence of the brittle fracture phase;
5. The obtained surface after machining with a conventional wheel showed significantly higher elevations in the roughness profile. When using a multigranular grinding wheel, the surface was characterized by aligned furrows, which was due to the unconventional design of the wheel, where smaller grains prevent the wheel from seizing and forming chips that damage the surface;
6. Use of a multigranular wheel resulted in a nearly 30% and 60% decrease in Sk and Skp values, respectively, when compared to conventional wheel;
7. Obtained results show that the multigranular wheel can be successfully used in grinding of difficult to cut materials such as Inconel 625. The construction of a new type of grinding tool makes it possible to conduct research to find the most adequate parameters of the grinding process. The ever-emerging new materials and the ever-increasing demands placed on machine parts are the perfect scenario for a number of implementations. A thorough exploration of the issue of grain contact in this configuration with the workpiece will make it possible to obtain better parameters of the machined surface;
8. In further studies, attention should be paid to the possibility of reducing the brittle fracture mechanism using a multigranular wheel in favor of plastic flow. The process should determine the parameters at which this is possible.

## Figures and Tables

**Table 1:** Chemical composition of Inconel 625 [38].

Element	Ni	Cr	Mo	Nb	Fe	Ti	C	Mn	Si
mass%	58	20–23	8–10	3.15–4.15	max 5	max 0.4	max 0.1	max 0.5	max 0.5

**Table 2:** Experimental conditions.

Workpiece	Inconel 625
Tool 1	01_250x32x76_IPA_80/100/120_HA_26VTX2 (abrasive grain size: 125–212 µm)
Tool 2	01_250x32x76_IPA_100_HA_26VTX2 (abrasive grain size: 150 µm)
Cutting speed (*Vc*)	7–34 (m/s)
Transverse feed speed (*Vp*)	90–300 (mm/min)
Longitudinal feed speed (*Vw*)	6000–16,000 (mm/min)
Depth of cut (ap)	0.04 (mm)
Number of passes (z)	3

**Table 3:** The ranges of the parameters used depending on the levels of α.

Level	Parameters		
	*Vc* (m/s)	*Vw* (mm/min)	*Vp* (mm/min)
−α	7	6000	90
−1	13	8030	132
0	22	11,000	195
1	31	13,900	257
α	34	16,000	300

**Table 4:** Abbott–Firestone curves (*Vw* = 11,000 mm/min, *Vp* = 90 mm/min and *Vc* = 23 m/s).

**Multigranular Wheel (Sa 1.0 μm)**		**Conventional Wheel (Sa 1.4 μm)**	
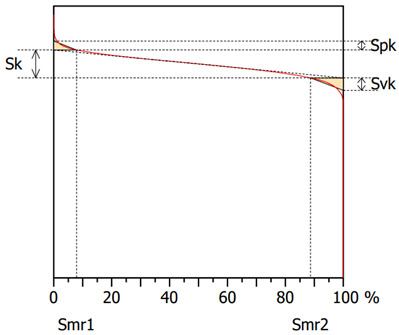		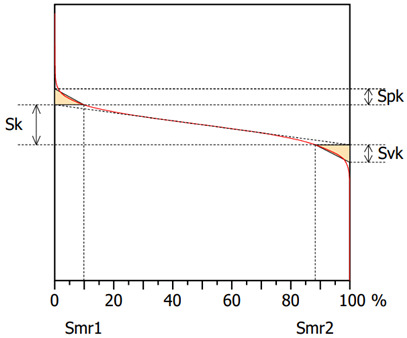	
**Parameter**	**Value**	**Parameter**	**Value**
Sk	3.5 μm	Sk	4.5 μm
Spk	1.1 μm	Spk	1.8 μm
Svk	1.6 μm	Svk	2 μm
Smr1	7.9%	Smr1	9.8%
Smr2	88.7%	Smr2	88.2%

**Table 5:** Results obtained for a conventional grinding wheel and a multigranular grinding wheel.

No	Input Parameters			Sa (μm)	
	*Vw* (mm/min)	*Vp* (mm/min)	*Vc* (m/s)	Conventional Wheel	Multigranular Wheel
1	8027	133	13	4.00	2.10
2	13,973	257	13	1.30	3.80
3	8027	257	33	1.40	1.40
4	13,973	133	33	0.95	1.00
5	11,000	195	23	1.40	1.70
6	8027	257	13	1.30	2.40
7	13,973	133	13	2.00	1.50
8	8027	133	33	1.20	0.90
9	13,973	257	33	1.40	1.30
10	11,000	195	23	1.50	1.20
11	11,000	195	7	3.40	3.80
12	11,000	195	39	1.40	1.20
13	6000	195	23	1.50	3.30
14	16,000	195	23	1.30	1.60
15	11,000	90	23	1.40	1.00
16	11,000	300	23	1.60	1.40
17	11,000	195	23	1.30	1.40

**Table 6:** Representative stereometric images obtained with multigranular and grinding wheels.

Multigranular Wheel	Grinding Wheel
*Vw* = 11,000 mm/min, *Vp* = 195 mm/min, *Vc* = 7 m/s	
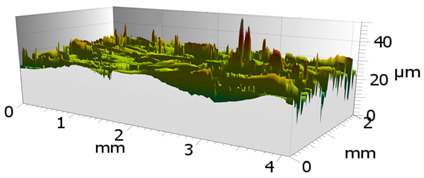	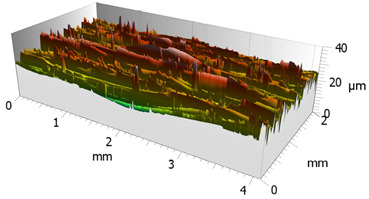
Sa = 3.4 µm	Sa = 3.8 µm
*Vw* = 13,973 mm/min, *Vp* = 133 mm/min, *Vc* = 13 m/s	
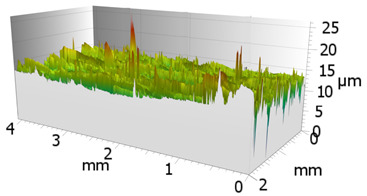	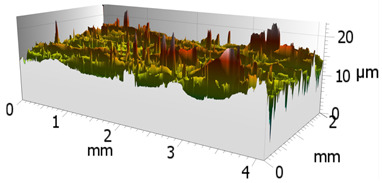
Sa = 1.50 µm	Sa = 2.00 µm
*Vw* = 8027 mm/min, *Vp* = 133 mm/min, *Vc* = 33 m/s	
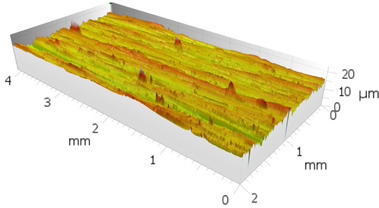	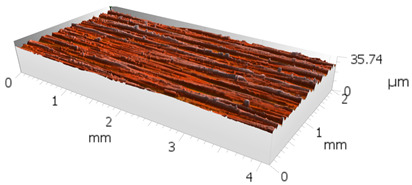
Sa = 0.9 µm	Sa = 1.2 µm

**Table 7:** Regression summary.

Figure Number	Wheel Type	R	R^2^	F/Fkr
Figures 6–8	Multigranular	0.87	0.75	6.6
Figures 9–11	Conventional	0.92	0.85	9.12

**Table 8:** ANOVA table for Sa (after elimination) for a conventional wheel.

Source	Sum of Squares	Degrees of Freedom	Mean Square	*F* Value	Prob > *f*	Contribution %
Model	8.7248	6	1.45413	2.53		
*Vw*	0.5089	1	0.5089	50.89	0.0190	5.83
*Vp*	0.4059	1	0.4059	40.59	0.0237	4.65
*Vc*	3.6288	1	3.6288	362.88	0.0027	41.59
*Vc* ^2^	1.4186	1	1.4186	141.86	0.0069	16.26
*VwVp*	0.6612	1	0.6612	66.12	0.0147	7.58
*VpVc*	2.1012	1	2.1012	210.12	0.0047	24.08
Error	0.5740	10				
Total SS	9.2988	16	**R-sqr = 0.85**		**R-Adj = 0.80**	

**Table 9:** ANOVA table for Sa (after elimination) for a multigranular wheel.

Source	Sum of Squares	Degrees of Freedom	Mean Square	*F* Value	Prob > *f*	Contribution %
Model	8.4641	4	1.454135	2.5		
*Vw*	0.3104	1	0.31045	93.1	0.0105	3.67
*Vc*	6.6775	1	6.67756	2003.2	0.0004	78.89
*Vc* ^2^	1.0711	1	1.07110	321.3	0.0030	12.65
*VwVp*	0.4050	1	0.40500	121.5	0.0081	4.78
Error	0.6848	12				
Total SS	9.2988	16	**R-sqr = 0.75**		**R-Adj = 0.69**	

## Data Availability

The data presented in this study are available on request from the corresponding authors. The data are not publicly available due to privacy.
